# A mouse model of inherited choline kinase β-deficiency presents with specific cardiac abnormalities and a predisposition to arrhythmia

**DOI:** 10.1016/j.jbc.2022.101716

**Published:** 2022-02-11

**Authors:** Mahtab Tavasoli, Tiam Feridooni, Hirad Feridooni, Stanislav Sokolenko, Abhishek Mishra, Abir Lefsay, Sadish Srinivassane, Sarah Anne Reid, Joyce Rowsell, Molly Praest, Alexandra MacKinnon, Melissa Mammoliti, Ashley Alyssa Maloney, Marina Moraca, Kitipong Uaesoontrachoon, Kanneboyina Nagaraju, Eric P. Hoffman, Kishore B.S. Pasumarthi, Christopher R. McMaster

**Affiliations:** 1 Department of Pharmacology, Dalhousie University, Halifax, Nova Scotia, Canada; 2 Department of Process Engineering & Applied Science, Dalhousie University, Halifax, Nova Scotia, Canada; 3 Mass Spectrometry Core Facility, Dalhousie University, Halifax, Nova Scotia, Canada; 4 Agada Biosciences Inc, Halifax, Nova Scotia, Canada; 5 School of Pharmacy and Pharmaceutical Sciences, Binghamton University, State University of New York (SUNY), Binghamton, New York, USA

**Keywords:** metabolism, lipid, phosphatidylcholine, acylcarnitine, muscular dystrophy, cardiac muscle, heart disease, AcCa, acyl carnitine, AMPK, AMP-activated protein kinase, ANP, atrial natriuretic peptide, BSA, bovine serum albumin, CHKA, choline kinase alpha, CHKB, choline kinase beta, CL, cardiolipin, DG, diacylglycerol, ERK, extracellular signal-regulated kinase, GSK-3β, glycogen synthase kinase-3β, ISO, isoproterenol, LPC, lysophosphatidylcholine, LV, left ventricular, OCR, oxygen consumption rate, PA, phosphatidic acid, p-AKT, phosphorylated AKT, PC, phosphatidylcholine, PE, phosphatidylethanolamine, PG, phosphatidylglycerol, TEM, transmission electron microscopy, TG, triacylglycerol, VCS, ventricular conduction system

## Abstract

The *CHKB* gene encodes choline kinase β, which catalyzes the first step in the biosynthetic pathway for the major phospholipid phosphatidylcholine. Homozygous loss-of-function variants in human *CHKB* are associated with a congenital muscular dystrophy. Dilated cardiomyopathy is present in some *CHKB* patients and can cause heart failure and death. Mechanisms underlying a cardiac phenotype due to decreased CHKB levels are not well characterized. We determined that there is cardiac hypertrophy in *Chkb*^−/−^ mice along with a decrease in left ventricle size, internal diameter, and stroke volume compared with wildtype and *Chkb*^+/−^ mice. Unlike wildtype mice, 60% of the *Chkb*^+/−^ and all *Chkb*^−/−^ mice tested displayed arrhythmic events when challenged with isoproterenol. Lipidomic analysis revealed that the major change in lipid level in *Chkb*^+/−^ and *Chkb*^−/−^ hearts was an increase in the arrhythmogenic lipid acylcarnitine. An increase in acylcarnitine level is also associated with a defect in the ability of mitochondria to use fatty acids for energy and we observed that mitochondria from *Chkb*^−/−^ hearts had abnormal cristae and inefficient electron transport chain activity. Atrial natriuretic peptide (ANP) is a hormone produced by the heart that protects against the development of heart failure including ventricular conduction defects. We determined that there was a decrease in expression of *ANP*, its receptor *NPRA*, as well as ventricular conduction system markers in *Chkb*^+/−^ and *Chkb*^−/−^ mice.

Human cell membranes separate and protect cells from their external environment, transmit cellular signals, establish an electrochemical gradient *via* the transport of ions, and generate action potential in neurons and muscle cells (1). The phospholipid bilayer is the fundamental structure of membranes and interacts with peripheral and integral membrane proteins to regulate critical biological processes (2). Phosphatidylcholine (PC) is the most abundant membrane phospholipid constituting between 30% and 60% of the phospholipid mass of eukaryotic cell membranes (3).

The first enzymatic step in the synthesis of PC is the phosphorylation of choline by choline kinases to produce phosphocholine. In humans and mice, two separate genes, *CHKA* and *CHKB*, encoding choline kinase alpha (CHKA) and choline kinase beta (CHKB), respectively, catalyze this reaction. In humans and mice autosomal recessive loss-of-function mutations in *CHKB* (*Chkb* in mouse) cause congenital muscular dystrophy with megaconial myopathy (OMIM: 602541) (4, 5). Clinical characteristics of *CHKB*-associated muscular dystrophy include delayed motor development, neonatal hypotonia, and intellectual disability without brain malformation. Variable cardiac phenotypes including dilated cardiomyopathy, decreased left ventricular systolic function, and congenital heart defects are reported in at least one-third of all known cases and remain a major reason of early death in affected individuals (5, 6, 7, 8, 9). How a cardiac phenotype is manifested owing to decreased CHKB level is not known.

Here we report that both homozygous and heterozygous *Chkb* mice have cardiomyopathy, decreased cardiac functional capacity, and increased susceptibility to cardiac arrhythmia. Alteration of cardiac lipid metabolism in *Chkb*^+/−^ and *Chkb*^−/−^ mice results in accumulation of the arrhythmogenic intermediary fatty acid metabolite acyl carnitine (AcCa). Furthermore, *Chkb*^+/−^ and *Chkb*^−/−^ mice displayed reduced expression of atrial natriuretic peptide (*ANP*) and its receptor (*NPRA*), along with specific defects in cellular signaling pathways known to enable heart function. Our data from *Chkb*-deficient mice provide mechanistic and observational insights into how a defect in PC synthesis can result in cardiac defects and may help explain similar phenotypes observed in *CHKB*-deficient patients.

## Results

### Loss of Chkb does not affect Chka level in heart

**Figure 1:**
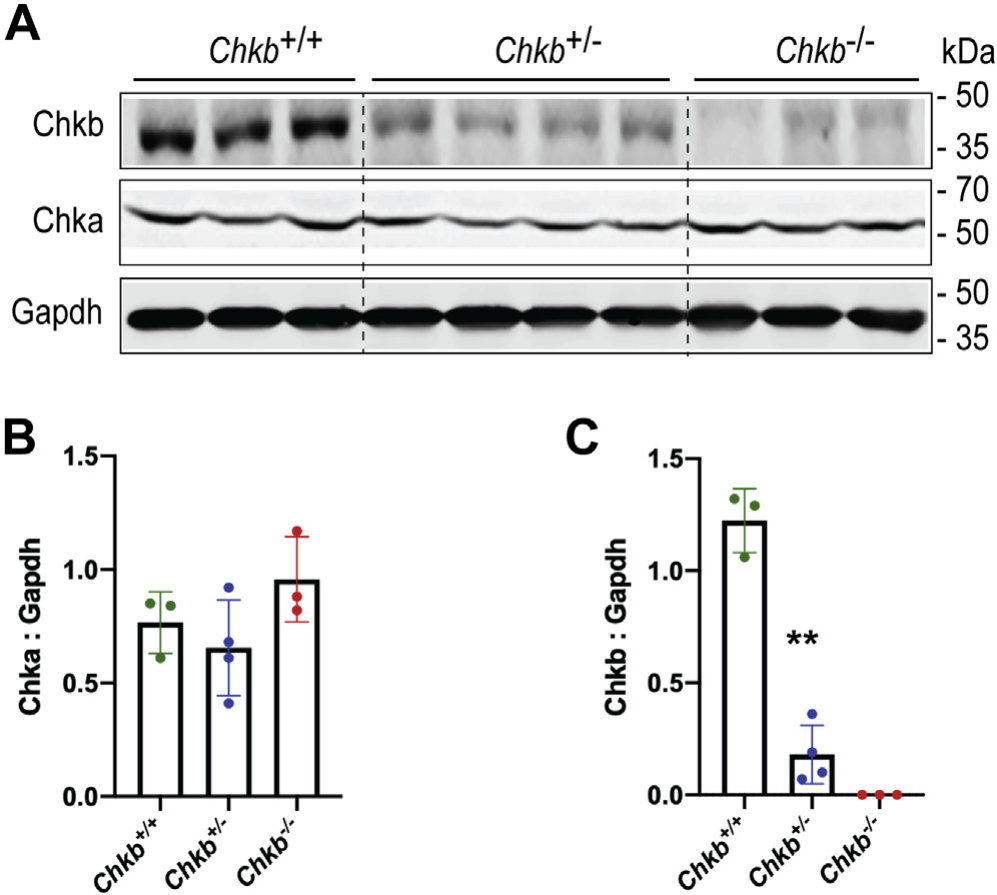
**Chka protein levels in heart samples from Chkb-deficient mice.***A*, Western blot of heart samples from three distinct (lanes 1–3) *Chkb*^*+/+*^, four distinct (lanes 4–7) *Chkb*^*+/*−^, and three distinct (lanes 8–10) *Chkb*^−*/*−^ mice probed with anti-Chkb, anti-Chka, and anti-Gapdh antibodies. *B* and *C*, densitometry of the Western blot data. Loss of *Chkb* does not affect Chka level in heart. Values are means ± SD; data were analyzed using one-way ANOVA with Tukey’s multiple comparison test. ∗∗*p*< 0.01 (n = 3–4 females per group).

*Chkb* encodes choline kinase β, the first enzymatic step in the synthesis of PC. A second choline kinase isoform, *Chka*, encoded by a separate gene, is present in mouse (and human) tissues. We investigated the protein expression pattern of these two choline kinase isoforms in cardiac muscle from *Chkb*^+/+^, *Chkb*^+/−^, and *Chkb*^−/−^ mice. Western blot analysis revealed that, as expected, Chkb protein expression is significantly reduced in *Chkb* heterozygous mice and is completely absent in homozygous knockout mice. Chka is expressed at a similar level in cardiac tissue from wildtype, *Chkb*^*+/*−^, and *Chkb*^*−/−*^ mice; no compensatory upregulation of Chka protein was observed in mice lacking the Chkb isoform (Fig. 1, *A*–*C*). The data are consistent with previous work that Chkb deficiency does not alter Chka mRNA expression in heart (4).

### Defective cardiac output due to decreased Chkb level

**Figure 2:**
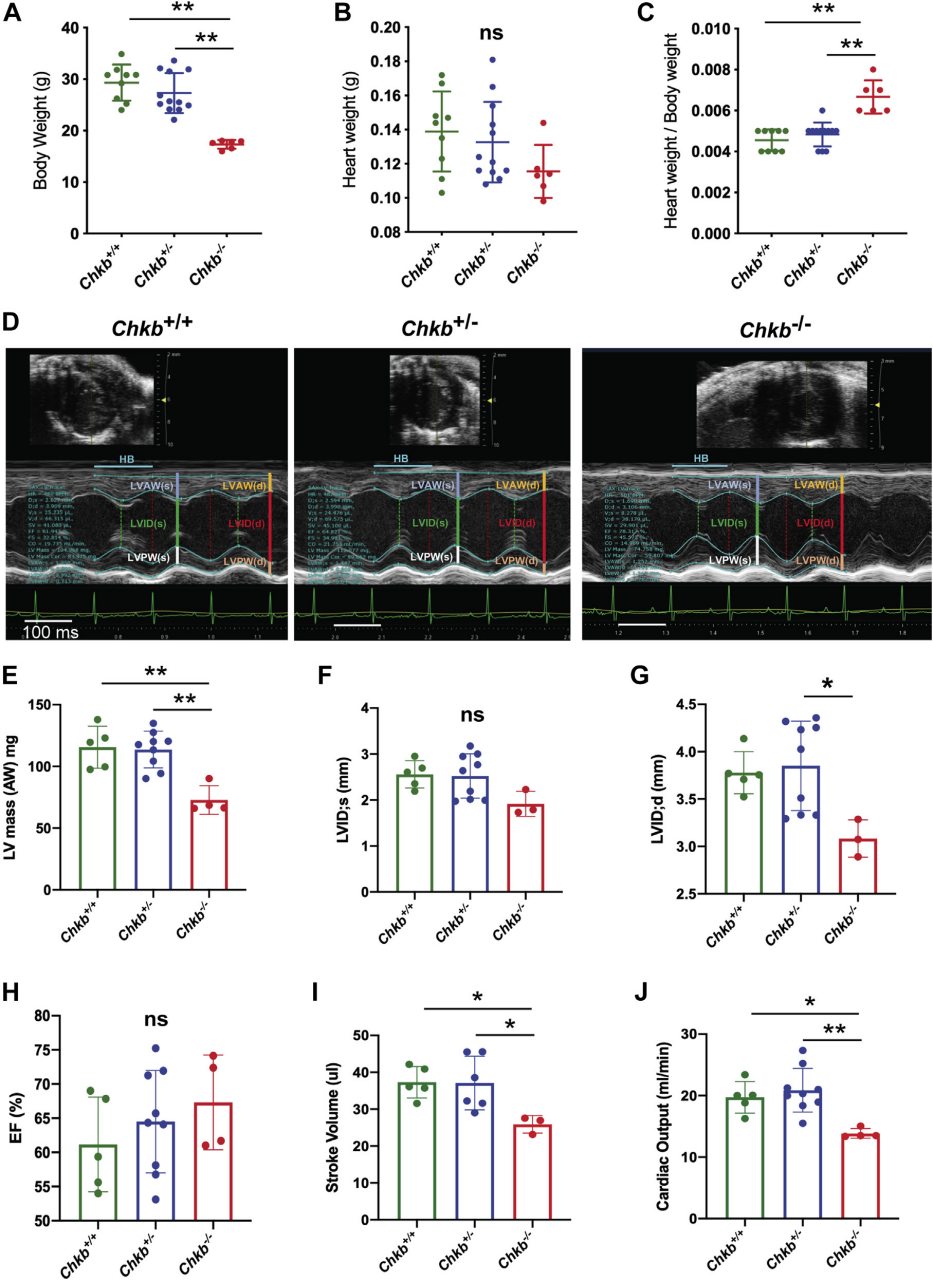
***Chkb* deficiency results in defects in heart function.***A*, body weight was recorded for 5-month-old *Chkb*^+/+^ (five male, four female), *Chkb*^+/−^ (four male, eight female), and *Chkb*^−/−^ (three male, three female) mice. Hearts were surgically removed from anesthetized mice and trimmed of extracardiac tissue. Heart weight was measured (*B*) and heart weight to body weight ratio (heart weight/body weight), an index of hypertrophy, was calculated (*C*). Values are presented as mean ± SD. Significance was calculated using one-way ANOVA with Tukey’s multiple comparison test for each specific time point. ∗∗*p* < 0.01 (n = 6–12 mice/group). *D*, representative M-mode images of 20-week-old *Chkb*^+/+^, *Chkb*^+/−^, and *Chkb*^−/−^ mice. *E*, left ventricle mass; LV Mass AW (mg). *F*, left ventricle internal diameter during systole; LVID;s (mm). *G*, left ventricle internal diameter during diastole; LVID;d (mm). *H*, ejection fraction (EF, %). *I*, stroke volume (μl) and (*J*) Cardiac output (ml/min). Data were analyzed using one-way ANOVA (*p* < 0.05) with Tukey’s multiple comparison test for each specific time point; ∗*p* < 0.05, ∗∗*p* < 0.01 (n = 3–12 mice/group). ns, not significant.

Echocardiography was used to assess systolic and diastolic function in hearts from 5-month-old *Chkb*^+/+^, *Chkb*^+/−^, and *Chkb*^−/−^ mice (Fig. 2 and Table 1). After cardiac function assessment, mouse body weight was determined and hearts were surgically removed from anaesthetized mice and heart weight was measured. *Chkb*^−/−^ mice weighed 40% less than *Chkb*^+/+^ or *Chkb*^+/−^ mice (Fig. 2*A*), whereas heart weight was similar among the three different genotypes. Heart weight to body weight ratio, an index of hypertrophy, was significantly increased in *Chkb*^−/−^ mice compared with the *Chkb*^+/+^ and *Chkb*^+/−^ mice (Fig. 2, *B* and *C*).

**Table 1:** Cardiac phenotypes of 5-month-old mice deficient in *Chkb*

Parameter	*Chkb*^+/+^ (n =5)	*Chkb*^+/−^ (n = 9)	*Chkb*^−*/*−^ (n = 3)	Significant
Gender (male; female)	(2; 3)	(3; 6)	(1; 2)	-
Heart rate (bpm)	504.88 ± 44.90	515.1 ± 60.0	551.38 ± 47.80	NS
M-Mode				
LVID (sys) (mm)	2.56 ± 0.30	2.52 ± 0.48	1.92 ± 0.27	NS
LVID(d) (mm)	3.78 ± 0.22	3.85 ± 0.47	3.08 ± 0.20^a^^,^^b^	*p* < 0.05
LVPW(sys) (mm)	1.14 ± 0.15	1.20 ± 0.26	1.11 ± 0.05	NS
LVPW(d) (mm)	0.87 ± 0.10	0.88 ± 0.16	0.87 ± 0.09	NS
LV Vol(sys) (μl)	24.14 ± 6.84	24.14 ± 10.89	11.73 ± 4.35	
LV Vol(d) (μl)	61.44 ± 8.82	65.28 ± 18.48	37.61 ± 5.85^a^	*p* < 0.05
Calculated stroke volume (μl)	37.3 ± 4.28	41.14 ± 8.75	25.88 ± 2.38^a^	*p* < 0.05
EF%	61.16 ± 6.92	64.51 ± 7.49	69.41 ± 6.73	NS
FS%	32.4 ± 4.9	34.91 ± 5.35	38.05 ± 5.12	NS
Calculated cardiac output (ml)	18.87 ± 3.09	20.87 ± 3.55	13.97 ± 0.92^a^	*p* < 0.05
LV mass AW (mg)	115.58 ± 16.98	113.66 ± 14.84	75.07 ± 13.15^a^^,^^b^	*p* < 0.01
LV mass AW (corrected) (mg)	92.47 ± 13.59	90.93 ± 11.87	60.06 ± 10.52^a^^,^^b^	*p* < 0.01
LVAW(sys) (mm)	1.29 ± 0.18	1.27 ± 0.12	1.09 ± 0.04	NS
LVAW(d) (mm)	0.82 ± 0.14	0.76 ± 0.10	0.71 ± 0.02	NS
Pulse-wave Doppler echocardiography				
MV E (m/s)	546.05 ± 72.17	607.41 ± 77.31	543.06 ± 63.45	NS
MV A (m/s)	454.86 ± 75.05	495.07 ± 86.35	413.80 ± 38.67	NS
MV E/A	1.21 ± 0.08	1.24 ± 0.16	1.32 ± 0.17	NS

Abbreviations: EF%, percent ejection fraction; FS%, percent fraction shortening; HET, heterozygous for *Chkb;* LVID(d), left ventricle internal diameter during diastole mm; LVID(sys), left ventricle internal diameter during systole; LV Vol(d), left ventricle volume during diastole; LV Vol(sys) (μl), left ventricle volume during systole; LV Mass AW (mg), left ventricle mass; LV Mass AW (Corrected), left ventricle mass; LVAW(d), left ventricle anterior wall during diastole; LVAW(sys) (mm), left ventricle anterior wall during systole; LVPW(d), left ventricle posterior wall during diastole; LVPW(sys), left ventricle posterior wall during systole; MVA, mitral valve active phase; MVE, mitral valve early phase; MVE/A, ratio of early to active phase.

a Significant from WT.

b Significant from HET.

Cardiac structural parameters were measured for *Chkb*^+/+^, *Chkb*^+/−^, and *Chkb*^−/−^ mice. Heart rate was not significantly different during echocardiogram measurements between each group (Table 1). There were no structural differences at diastole and systole in hearts from *Chkb*^+/+^ and *Chkb*^+/−^ mice (Fig. 2 and Table 1); however, *Chkb*^−/−^ mice had a smaller left ventricular (LV) mass and smaller LV internal diameter during diastole (Fig. 2, *E*–*G*).

In terms of functional parameters, there were no differences between *Chkb*^+/+^ and *Chkb*^+/−^ mice, whereas *Chkb*^−/−^ mice had significantly smaller calculated stroke volume (Fig. 2*I*), calculated cardiac output (Fig. 2*J*), and a reduction in LV volume during diastole (Table 1). We also measured diastolic function using pulse-wave Doppler echocardiography. There were no significant differences in mitral valve, E wave, A wave, and the E-to-A ratio between groups (Table 1). Together, these findings suggest that a complete loss of Chkb reduces LV mass, internal diameter during diastole, LV volume during diastole, and stroke volume and cardiac output and increases cardiac hypertrophy.

### Chkb-deficient mice are more susceptible to ventricular arrhythmia

**Figure 3:**
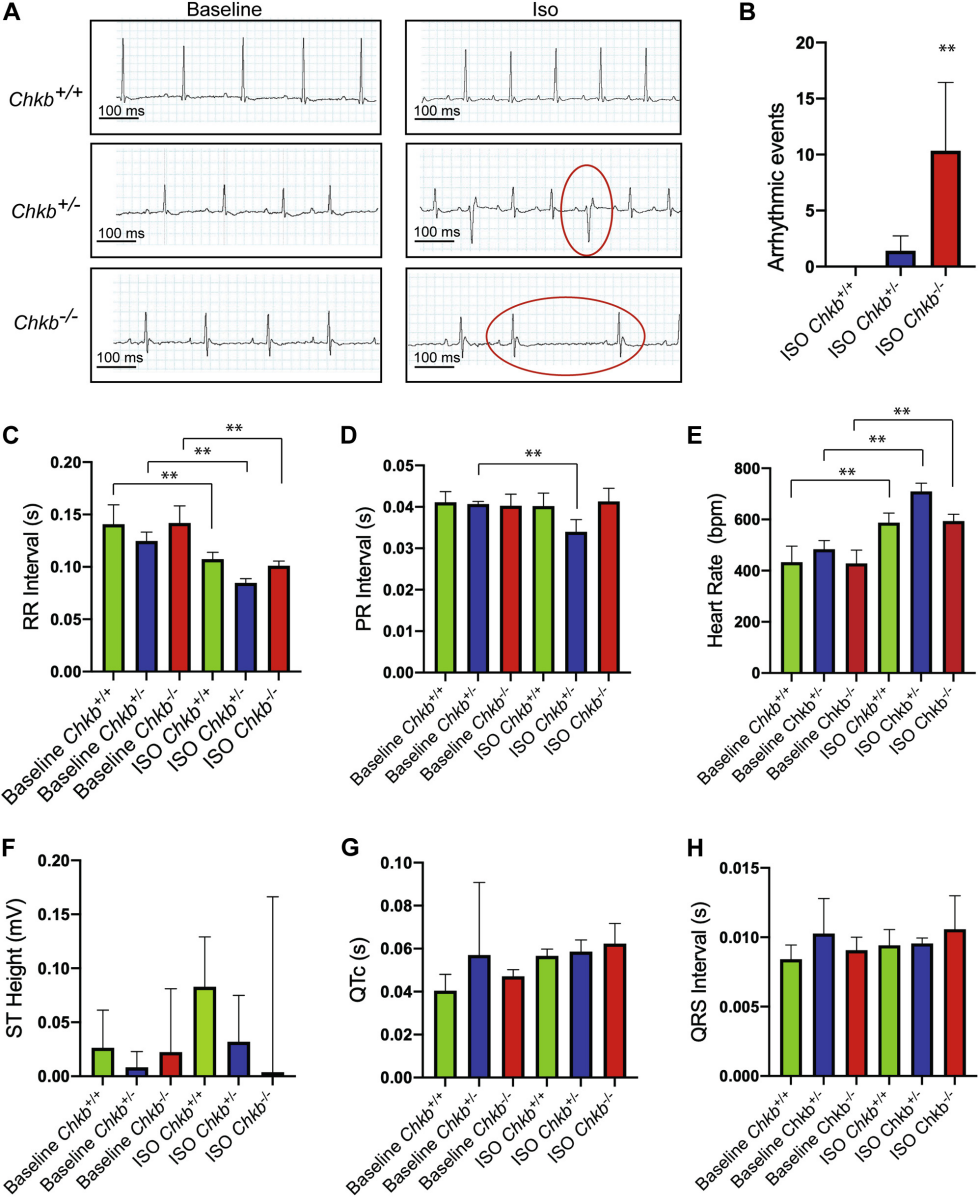
**Increased arrhythmic events due to *Chkb* deficiency.***A*, lead II ECG trace pattern of *Chkb*^*+/+*^ mice showing regular ECG pattern with defined P, QRS, and T waves at baseline and after isoproterenol (ISO). *Chkb*^*+/*−^ and *Chkb*^*−/−*^ mice showing regular ECG pattern at baseline but display arrhythmic events when challenged with ISO (highlighted with *red circles*). Quantification of arrhythmic events over 15 min (*B*) RR intervals (*C*), PR interval (*D*), heart rate (bpm) (*E*), ST height (mV) (*F*), QTc (s) (*G*), and QRS intervals (s) (*H*) at baseline and after treatment with ISO. Each bar represents mean ± SD. Data were analyzed using one-way ANOVA with Tukey’s multiple comparisons post hoc test; ∗∗*p*< 0.01 (n = 5–9 mice per group).

To determine if there were additional cardiac anomalies associated with decreased Chkb level, we evaluated mouse heart function by electrocardiogram (ECG) at baseline and when challenged with isoproterenol (ISO), a β1 and β2 adrenergic receptor agonist whose administration can reveal increased predilection toward arrhythmia. ECG measurements obtained from *Chkb*^+/+^, *Chkb*^+/−^, and *Chkb*^−/−^ mice determined that none of the mice displayed arrhythmic events at baseline. ISO treatment did not induce arrhythmic events in wildtype mice (Fig. 3*A*); ISO treatment resulted in 60% of the *Chkb*^+/−^ mice and 100% of *Chkb*^−/−^ mice exhibiting arrhythmic events (Fig. 3, *A* and *B*). Quantification of the baseline ECG parameters did not show a significant difference in RR intervals (Fig. 3*C*), PR interval (Fig. 3*D*), heart rate (Fig. 3*E*), ST height (Fig. 3*F*), QTc (Fig. 3*G*), or QRS interval (Fig. 3*H*) between *Chkb*^+/+^, *Chkb*^+/−^, and *Chkb*^−/−^ mice. When animals were treated with ISO, there was a significant decrease in RR interval in all genotypes compared with baseline RR intervals (Fig. 3*C*); however, there were no significant differences between RR intervals of animals that were stimulated with ISO (Fig. 3*C*). Furthermore, when *Chkb*^+/+^, *Chkb*^+/−^, and *Chkb*^−/−^ mice were challenged with ISO, only the PR interval of *Chkb*^+/−^ mice showed a significant reduction (Fig. 3*D*). There were no significant changes in ST height (Fig. 3*F*), QTc (Fig. 3*G*), and QRS interval (Fig. 3*H*) after treatment with ISO. In summary, infusion of ISO had little effect on ECG parameters but had a significant increase in arrhythmic events in *Chkb*-deficient mice.

### Chkb deficiency alters the lipid profile of the heart

**Figure 4:**
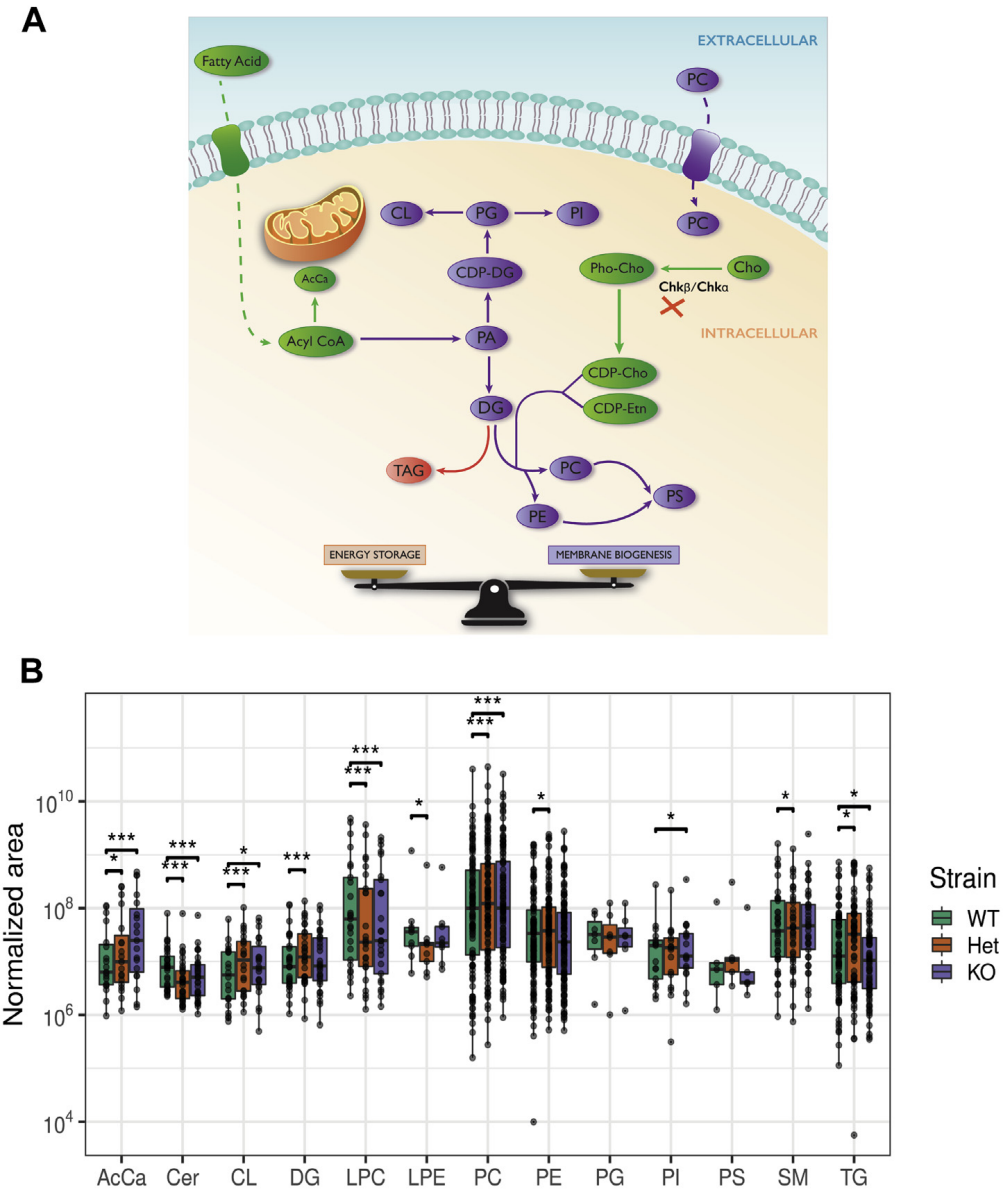
***Chkb* deficiency alters the cardiac lipid profile of *Chkb***^***+/*−**^**and *Chkb***^**−*/*−**^**mice and decreases mitochondrial β-oxidation capacity.***A*, PC synthesis is integrated with the synthesis of other major phospholipid classes, as well as AcCa, fatty acids, and the neutral lipids diacylglycerol (DG) and triacylglycerol (TG). *B*, comparison of expression levels of major glycerophospholipids (PC, PE, PI, PG, PS, CL, DG, and TG) and their metabolites (LPC, LPE) as well as sphingomyelin (SM), ceramide (Cer), and acyl carnitine (AcCa) between *Chkb*^*+/+*^, *Chkb*^*+/*−^, and *Chkb*^−*/*−^ mice (n = 3 mice per group). The analysis was performed on 5-month-old heart samples. Each *dot* represents an individual fatty acyl lipid species, and the bar represents total mass. All statistical comparisons were performed pair-wise and log scaled. That is, a fold-change was calculated between the *Chkb*^*+/*−^ and *Chkb*^−*/*−^ mice and controls for each unique lipid species, and a nonparametric statistical test was used to assess if the fold-changes were statistically significant from 1 in aggregate. There were no statistically significant changes for unique lipid species, but were so for total mass. Wilcoxon signed rank test with Bonferroni correction was used to determine the significance of a median pair-wise fold-increase in lipid amounts at an overall significance level of 5%. As the Bonferroni correction is fairly conservative, significant differences are reported at both precorrection (∗) and postcorrection (∗∗∗) significance levels. AcCa, acylcarnitine; CDP, cytidine diphosphate; Cer, ceramide; Cho, choline; CL, cardiolipin; DG, diacylglycerol; Etn, ethanolamine; LPC, lysophosphatidylcholine; LPE, lysophosphatidylethanolamine; PA, phosphatidic acid; PC, phosphatidylcholine; PE, phosphatidylethanolamine; PG, phosphatidylglycerol; Pho, phospho; PI, phosphatidylinositol; PS, phosphatidylserine; SM, sphingomyelin; TG, triacylglycerol.

PC synthesis is integrated with the synthesis of other major phospholipid classes, as well as AcCa, fatty acids, and the neutral lipids diacylglycerol (DG) and triacylglycerol (TG) (Fig. 4*A*). Fatty acids derived from plasma are first activated by esterification to fatty acyl-CoA. Subsequently, they will either be converted to AcCa to be used as energy by mitochondrial β-oxidation or be funneled into phosphatidic acid synthesis that can either enter the cytidine diphosphate diacylglycerol (CDP-DG) pathway to generate phosphatidylinositol, phosphatidylglycerol (PG), and cardiolipin (CL) or be converted to DG. DG can be metabolized into the two most abundant membrane phospholipids, PC and phosphatidylethanolamine (PE), or be used for the production of TG where it forms cytosolic lipid droplets. Lipidomics was used to determine if complete or partial loss of Chkb function alters lipid levels in heart. In cardiac muscle isolated from *Chkb*^*+/+*^, *Chkb*^*+/*−^, and *Chkb*^−*/*−^ mice the levels of the major glycerophospholipids, PC, PE, CL, PG, PI, PS, the neutral lipids, TG and DG, the sphingolipids, sphingomyelin and ceramide, the PC and PE metabolites, lysophosphatidylcholine (LPC), lysophosphatidylethanolamine, as well as acylcarnitine (AcCa) were quantified (Fig. 4*B* and Table 2). By far the largest change observed in the cardiac lipid profile was a 3.1-fold increase in AcCa level in cardiac muscle from *Chkb*^−*/*−^ mice and a 1.7-fold increase in *Chkb*^*+/*−^ mice, compared with wildtype. Small but statistically significant changes were also observed in several lipid species including a small increase in PC of 1.18- and 1.35-fold (Table 2) and a significant decrease in LPC to 0.62 and 0.52 in the *Chkb*^*+/*−^ and *Chkb*^−*/*−^ mice, respectively. The level of PC was not found to be significantly different in heart and other organs in previous work (4); this may be due to either differences in methods used or the fact that the previous study used 1-month-old mice, whereas the mice used in the current study were 5 months of age, a stage where the disease is much more advanced.

**Table 2:** Fold change in lipid levels due to *Chkb* deficiency

Lipid	Fold-change *Chkb*^+/−^/*Chkb*^+/+^	*p*-value	Significance	Fold-change *Chkb*^−/−^/*Chkb*^+/+^	*p*-value	Significance
AcCa	1.68	0.0053	∗	3.07	0.0002	∗∗∗
Cer	0.60	0.0000	∗∗∗	0.65	0.0000	∗∗∗
CL	1.67	0.0000	∗∗∗	1.40	0.0074	∗
DG	1.37	0.0000	∗∗∗	1.05	0.5291	
LPC	0.62	0.0001	∗∗∗	0.52	0.0007	∗∗∗
LPE	0.54	0.0039	∗	0.79	0.2500	
PC	1.18	0.0000	∗∗∗	1.35	0.0000	∗∗∗
PE	1.24	0.0071	∗	1.04	0.7155	
PG	0.64	0.8125		1.21	1.0000	
PI	0.87	0.7819		1.24	0.0174	∗
PS	1.74	0.0625		0.88	0.6250	
SM	0.82	0.0027	∗	1.06	0.3777	
TG	1.19	0.0418	∗	0.55	0.0155	∗

Pairwise Wilcoxon signed rank test with Bonferroni correction was used to determine the significance of a median pair-wise fold-increase in lipid amounts at an overall significance level of 5%. As the Bonferroni correction is fairly conservative, significant differences are reported at both precorrection (∗) and postcorrection (∗∗∗) significance levels. N = 3 mice per genotype.

Abbreviations: AcCa, acyl carnitine; Cer, ceramides; CL, cardiolipin; DG, diglyceride; LPC, lysophosphatidylcholine; LPE, lysophosphatidylethanolamine; PC, phosphatidylcholine; PE, phosphatidylethanolamine; PG, phosphatidylglycerol; PI, phosphatidylinositol; PS, phosphatidylserine; SM, sphingomyelin; TG, triglyceride.

Increases in two lipids are known to cause arrhythmia, AcCa and LPC. The level of LPC decreased in *Chkb*^−*/*−^ mice, whereas that of AcCa was substantively higher. We suggest that the dramatic increase in AcCa predisposes *Chkb*^*+/*−^ and *Chkb*^−*/*−^ mice to cardiac arrhythmia (10, 11, 12, 13, 14, 15, 16, 17, 18, 19, 20, 21).

### Defective cardiac mitochondrial β-oxidation due to Chkb deficiency

**Figure 5:**
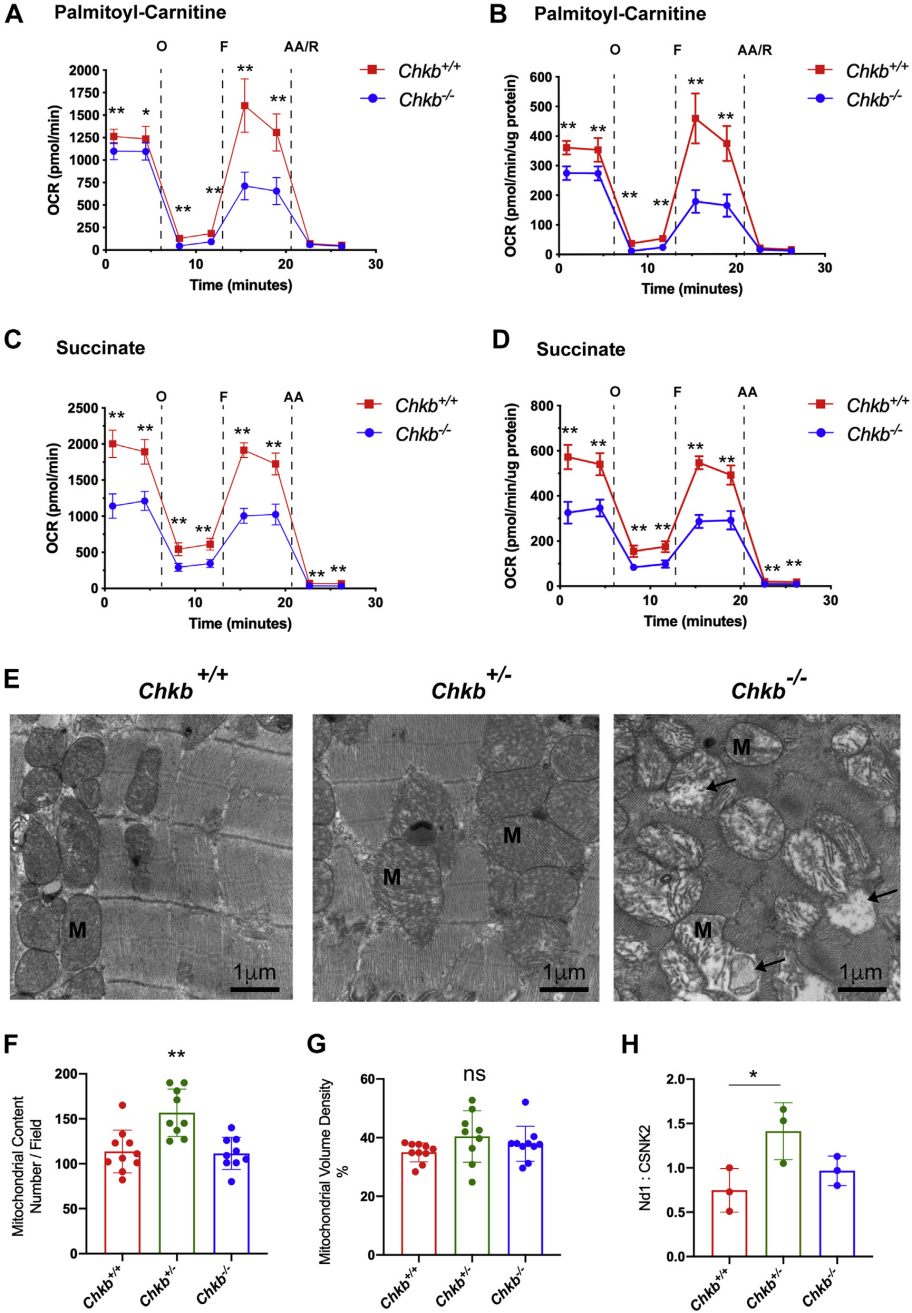
**Defective cardiac mitochondrial β-oxidation and altered mitochondrial morphology due to Chkb deficiency.** Coupled mitochondrial respiration assay tracings as determined by multiwell measurement of oxygen consumption driven by 40 μM palmitoylcarnitine/1 mM malate (*A* and *B*) or 10 mM succinate/2 μM rotenone (*C* and *D*). ADP (4 mM) was included in the initial media. Oxygen consumption rate (OCR) values are shown before (*A* and *C*) and after normalization to total protein (*B* and *D*). Each well contains 3.5 to 4 μg total protein (updated in the normalization settings). To inhibit coupled respiration, oligomycin A (a complex V inhibitor) is added to the mitochondria. FCCP is a mitochondrial uncoupler, allowing protons to cross the inner mitochondrial membrane, and induces uncoupled respiration, circumventing complex V. Finally, antimycin A, an inhibitor of complex III, is added to assess nonmitochondrial respiration. Values are means ± SD. Analysis performed by two tailed Student's *t* test for each time point; ∗∗*p*< 0.01 (n = 4–5 wells per group). *E*, ultrastructural changes in the mitochondrial membrane in cardiac tissue. TEM appearance of the mitochondrial profile of hearts from 30-day-old *Chkb*^*+/+*^, *Chkb*^*+/*−^, and *Chkb*^−*/*−^ mice (representative of three mice per group). Occasional mitochondrial cristae deformation with balloon expansion (*arrows*). Mitochondrial content (number per field) (*F*) and mitochondrial volume density (*G*) quantified by standard stereological analysis of TEM images at 10,000× magnification. *H*, relative mitochondrial Nd1 gene expression. Data were analyzed using one-way ANOVA with Tukey’s multiple comparison test. ∗*p* < 0.05, ∗∗*p* < 0.01. For image analysis, three to four images per mice were used. n = 3 mice per group. AA, Antimycin A; F, Carbonyl cyanide-*4*- (trifluoromethoxy) phenylhydrazone (FCCP); M, mitochondria; O, Oligomycin A; R, Rotenone.

Fatty acids are the main energy substrate of the heart and provide most cofactors necessary for mitochondrial oxidative phosphorylation (22). Since an increase in AcCa can be a result of a decreased ability of mitochondria to uptake and use fatty acids as source of energy, palmitoyl-carnitine was used as an energy source to determine *β*-oxidation capacity in wildtype and *Chkb*^−*/*−^ mice. Intact/respiring cardiac mitochondria were isolated, and the oxygen consumption rate (OCR) of isolated mitochondria was measured using a Seahorse XF24 extracellular flux analyzer. We included saturating concentration of ADP to ensure the maximal state three rate for the duration of the measurement cycle, and sequential injections of oligomycin, FCCP, and antimycin A/rotenone followed. Mitochondria from *Chkb*^−/−^ heart showed significantly lower OCR in all respiratory states compared with mitochondria isolated from wildtype heart when using palmitoyl-carnitine as substrate (Fig. 5, *A* and *B*).

It is known that mitochondrial respiratory enzyme activities are dependent on membrane phospholipid composition (23); therefore, we tested if the decrease in OCR in *Chkb*^−/−^ cardiac mitochondria is fatty acid specific or is due to a general defect in function of the electron transport chain in the heart. We assessed respiration in wildtype and *Chkb*^−/−^ cardiac mitochondria using succinate, which feeds directly into the electron transport system through complex II. Rotenone (a complex I inhibitor) was used with succinate to prevent any complex I–driven respiration. Similar to fatty acid–driven OCR, when succinate was used as energy source, the *Chkb*^−/−^ hearts showed significantly lower OCR in all respiratory states determined compared with mitochondria isolated from wildtype mice (Fig. 5, *C* and *D*). There appears to be a general defect in electron transport chain function in cardiac muscle of *Chkb*^−/−^ mice.

### Morphological changes in mitochondria in Chkb-deficient heart

The electron transport chain is present on the inner mitochondrial membrane, and we determined that cardiac muscle from *Chkb*^−/−^ mice have decreased electron transport chain efficacy. To assess if mitochondrial morphological changes are present in hearts from *Chkb*-deficient mice, we examined cardiac mitochondrial morphology by transmission electron microscopy. Electron microscopic examination of heart muscles from wildtype and *Chkb*-deficient mice revealed mitochondria with cristae (inner mitochondrial membrane) deformation and reduced cristae density in *Chkb*^+/−^ mice, with cristae deformation and lack of density further exacerbated in the *Chkb*^−/−^ mice (Fig. 5*E*). To further evaluate mitochondria in cardiac muscle of *Chkb*-deficient mice, we employed stereological methods (24). The number of mitochondria in heart samples from *Chkb*^+/−^ mice is 38% more than that of the wildtype (Fig. 5*F*), whereas the percentage of cardiac volume occupied by mitochondria was similar among the different groups (Fig. 5*G*). Consistent with the stereological analysis, the mitochondrial gene expression of NADH-ubiquinone oxidoreductase chain 1 (*MT-Nd1*) in *Chkb*^+/−^ mouse cardiac muscle was increased 1.9-fold (Fig. 5*H*). The increase in abnormal cristae is consistent with the overall decrease in electron transport chain function in *Chkb*^−/−^ mice.

### Reduced gene expression of ANP, ANP receptor, and conduction system markers in Chkb-deficient mice

**Figure 6:**
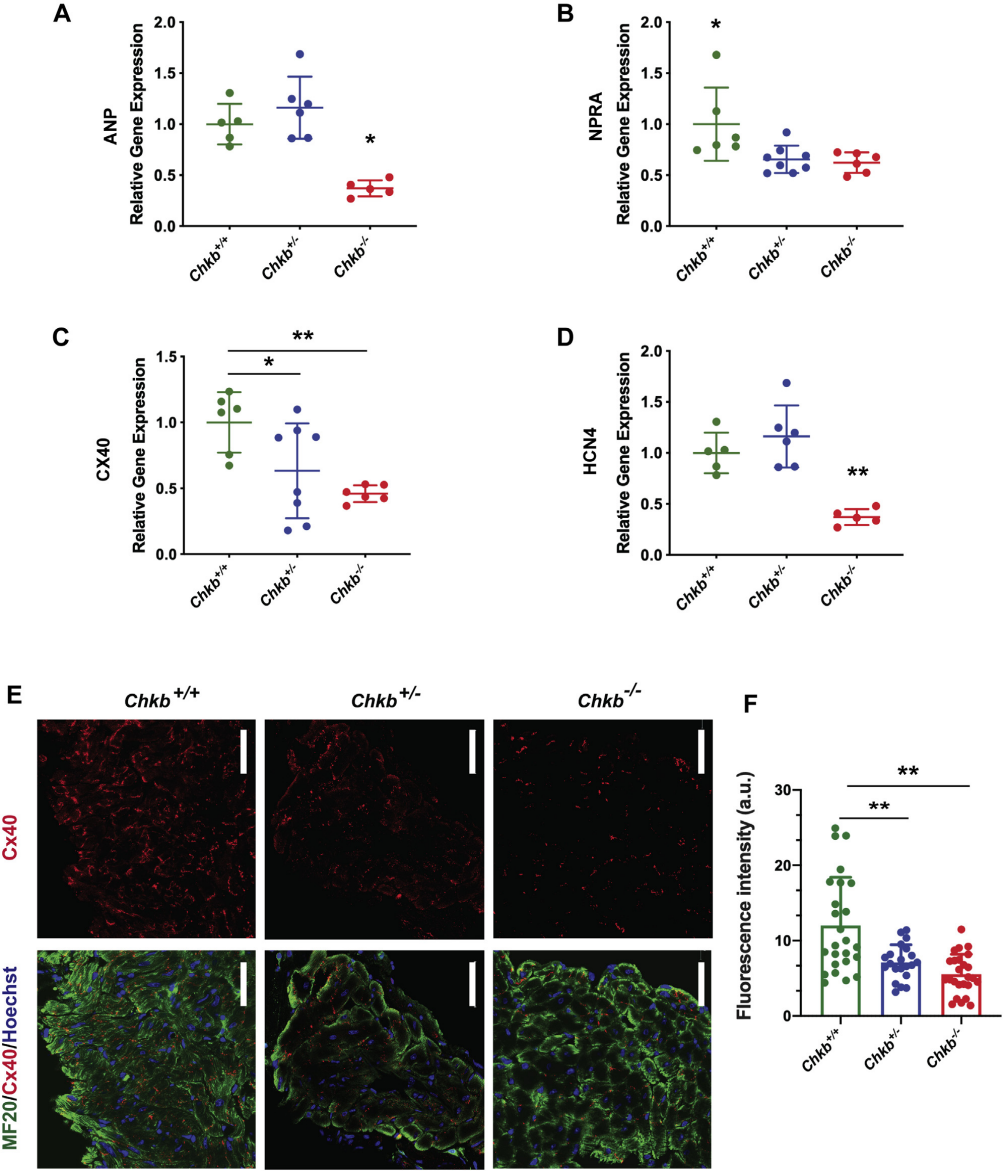
**Reduced expression of cardiac conduction system markers in *Chkb*-deficient mice.** RT–quantitative PCR analysis was used to monitor gene expression of atrial natriuretic peptide (*ANP*) (*A*), natriuretic peptide receptor-A (*NPRA*) (*B*), ventricular conduction system markers connexin 40 (*Cx40*) (*C*), and hyperpolarization-activated cyclic nucleotide-gated channel-4 (*HCN4*) (*D*). Expression levels were normalized to *Gapdh via* the ΔΔC_T_ method. n = 3 to 6 mice per group, each bar represents mean ± SD, ∗*p* < 0.01, ∗∗*p*< 0.01; one-way ANOVA with Tukey’s multiple comparisons post hoc test. *E* and *F*, representative images and quantitation of cardiac muscle sections of 30-day-old *Chkb*^*+/+*^, Chkb^*+/*−^, and *Chkb*^−*/*−^ mice stained with sarcomeric myosin MF20 (*green*) and Cx40 antibodies (*red*) along with a Bodipy nuclear stain. The scale bar represents 50 μM.

Dilated cardiomyopathy is the most commonly reported cardiac phenotype in *CHKB* patients (5, 6) and is a frequent cause of heart failure and death (25). ANP is a cardiac hormone considered an accurate biomarker of dilated cardiomyopathy (26, 27, 28, 29, 30). ANP exerts its physiological action *via* the NPRA receptor. RT–quantitative PCR showed that *ANP* expression is significantly reduced in heart from *Chkb*^−/−^ mice (Fig. 6*A*). Also, in both the heterozygous and homozygous state for *Chkb* there was reduced expression of *NPRA* (Fig. 6*B*). We have previously shown that ANP increases the expression of ventricular conduction system (VCS) markers in embryonic ventricular cells and NPRA deficiency leads to defects in Purkinje fiber arborization (27); therefore, we determined if the expression of the VCS markers hyperpolarization-activated cyclic nucleotide-gated channel-4 (*HCN4*) and connexin 40 (*Cx40*) are affected in hearts from *Chkb*-deficient mice. Partial or complete loss of *Chkb* significantly decreased *Cx40* expression when compared with wildtype (4-fold and 3.4-fold, respectively, Fig. 6*C*), whereas complete loss of Chkb also significantly reduced expression of *HCN4* (Fig. 6*D*). Cx40 visualization has been widely used to monitor development and maturation of the VCS (27, 31). To assess whether Chkb deficiency altered the development and maturation of the VCS we stained cardiac tissue with Cx40 antibody. Consistent with reduced *Cx40* gene expression, the Cx40 signal was noticeably decreased in *Chkb*^+/−^ compared with the wildtype and the complete loss of *Chkb* activity further reduced Cx40 signal intensity (Fig. 6, *E* and *F*). Decreased Chkb level results in a decrease in the levels of regulators and biomarkers for dilated cardiomyopathy and the development of the VCS.

### Decreased p-AKT, p-GSK3β, and p-AMPK expression in Chkb-deficient hearts

Membrane lipid composition is known to regulate cell signaling by associating with integral membrane receptors and influencing their function either through direct binding or by affecting membrane dynamics (32, 33). We assessed if known signaling pathways that affect heart function, namely, the AKT, GSK-3β, ERK, and AMPK pathways, were defective due to reduced Chkb level (Fig. 4, *A*–*I*).

**Figure 7:**
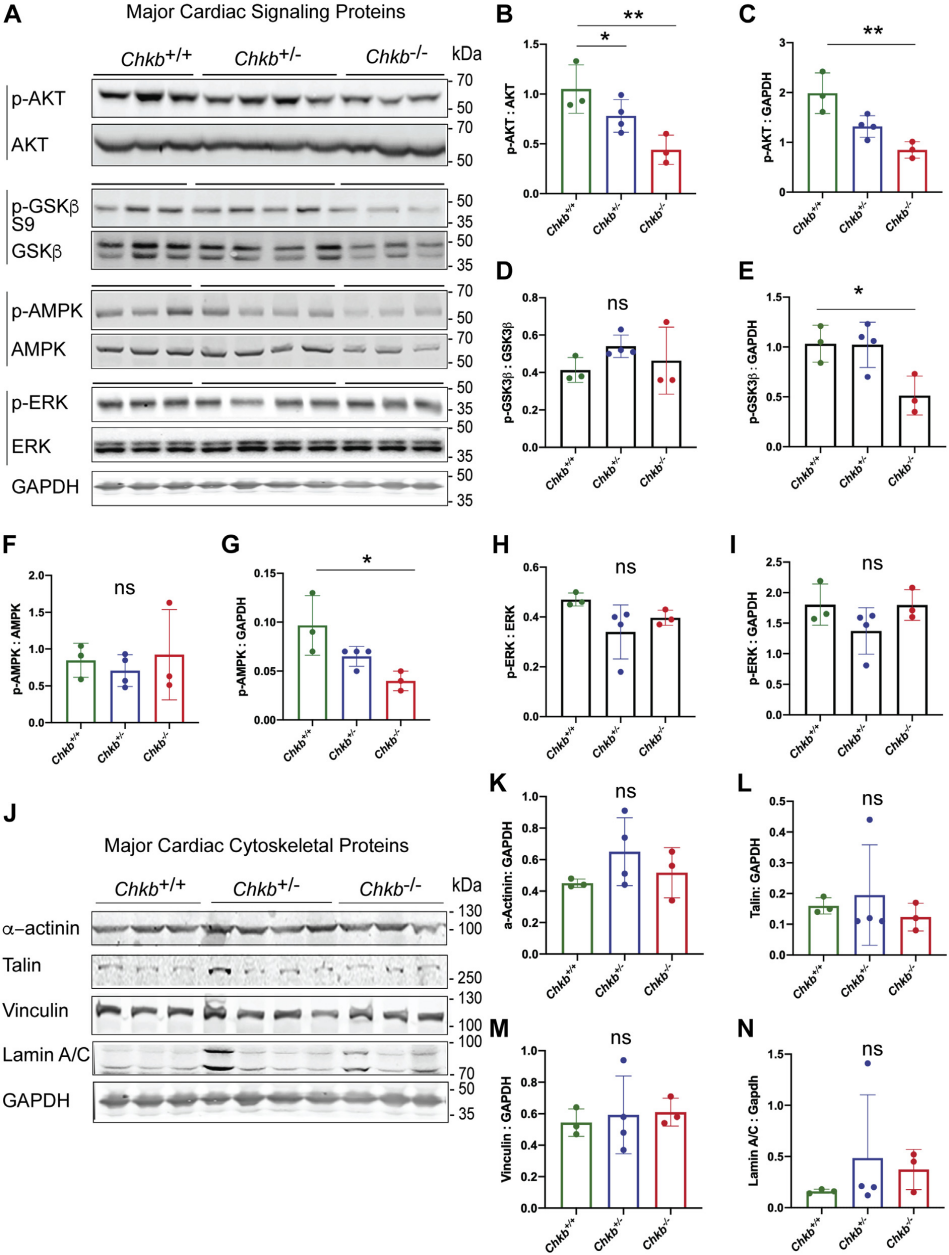
**Alterations in specific cardiac signaling pathways in *Chkb*-deficient hearts.***A*, Western blot of heart samples from three distinct (lanes 1–3) *Chkb*^*+/+*^, four distinct (lanes 4–7) *Chkb*^*+/*−^, and three distinct (lanes 8–10) *Chkb*^−*/*−^ mice probed with anti-p-AKT, anti-AKT, anti-p-GSK3β S9, anti-GSK3β, anti-p-AMPK, anti-AMPK, anti-p-ERK, anti-ERK, and anti-Gapdh antibodies. *B*–*I*, densitometry of the Western blot data. *J*, Western blot of heart samples from three distinct (lanes 1–3) *Chkb*^*+/+*^, four distinct (lanes 4–7) *Chkb*^*+/*−^, and three distinct (lanes 8–10) *Chkb*^−*/*−^ mice probed with major cytoskeletal proteins: anti-α-actinin, anti-Talin, anti-Vinculin, anti-Lamin A/C, and anti-Gapdh antibodies. *K*–*N*, densitometry of the Western blot data. Values are means ± SD. Data were analyzed using one-way ANOVA with Tukey’s multiple comparison test. ∗*p* < 0.01, ∗∗*p*< 0.01. n = 3 to 4 per group.

AKT signaling protects cardiomyocytes in both acute and chronic models of cardiac injury (34, 35, 36). Phosphorylation is the most important posttranslational determinant of AKT activity. One of the known downstream effectors of AKT, glycogen synthase kinase-3β (GSK-3β), negatively regulates cardiac hypertrophy. Inactivation of endogenous GSK-3β *via* phosphorylation is predominantly through the AKT pathway. We show that, in cardiac lysates from *Chkb*^−/−^ mice, phosphorylated AKT (p-AKT) and phosphorylated GSK-3β (p-GSK-3β) abundance decrease significantly relative to wildtype mice (Fig. 7, *A*–*E*). The decrease in p-GSK-3β was due to a decrease in total GSK-3β abundance, whereas the decrease in p-AKT reflected an absolute decrease in AKT phosphorylation (Fig. 7, *A*–*E*).

AMP-activated protein kinase (AMPK) is a key regulator of the metabolism of both fatty acids and glucose in the heart (37). The activation of AMPK by phosphorylation during metabolic stress is known to increase energy production and inhibit apoptosis. We determined if AMPK phosphorylation contributes to the cardiomyopathy in Chkb-deficient mice. In cardiac lysates from *Chkb*^−/−^ mice, phosphorylated AMPK abundance decreased significantly relative to *Chkb*^+/+^ mice (Fig. 7, *A* and *F*–*G*). The decrease in p-AMPK was due to a decrease in total AMPK abundance (Fig. 7, *A* and *F*–*G*).

Extracellular signal-regulated kinase (ERK) is activated downstream of G protein–coupled receptors and integrin stimulation and is involved in adaptive remodeling during the early phase of chronic pressure overload or maladaptive physiological changes during hypertension and chemotherapy-mediated cardiac side effects (38). Hetero- or homozygous deficiency in *Chkb* did not alter ERK expression or activation by phosphorylation in hearts (Fig. 7, *A* and *H*–*I*).

Dynamics and localization of specific lipid species, as well as membrane rigidity in living cells, can effect dynamic changes to the cytoskeleton, thereby impacting cell physiology. We tested if Chkb level affects the protein expression of some of the most important cardiac cytoskeletal proteins. Chkb deficiency did not alter the expression of the cytoskeletal proteins α-actinin, Talin, Vinculin, or Lamin A/C (Fig. 7, *J*–*N*).

Chkb deficiency decreases p-AKT, p-GSK3β, and p-AMPK levels in heart but has no effect on ERK signaling or cardiac cytoskeletal protein expression. These deficiencies in specific cell signaling pathways required for cardiac health and function point to specific, *versus* general, effects on signaling pathways due to the membrane lipid perturbations observed in mice deficient in Chkb.

## Discussion

**Figure 8:**
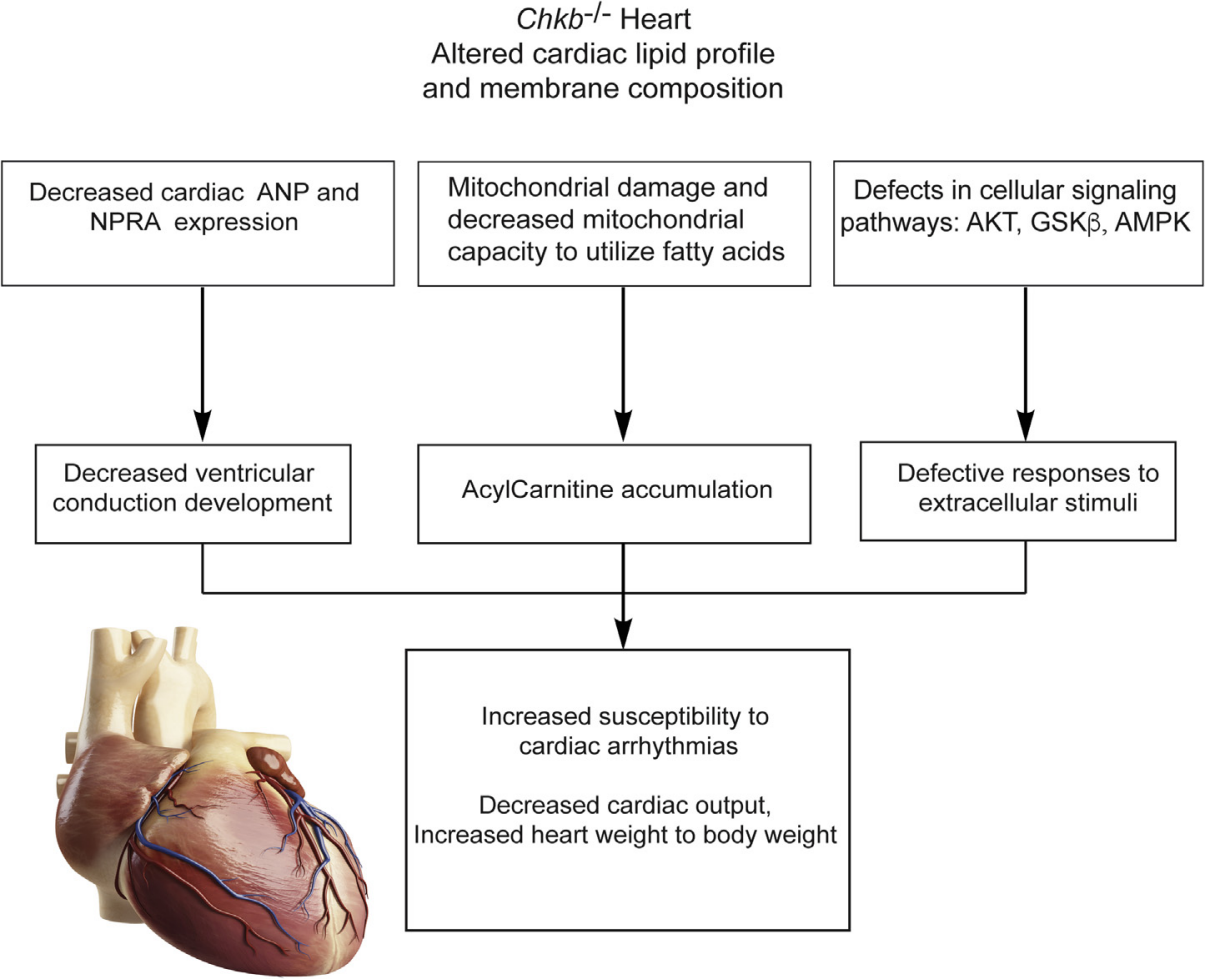
**Summary of cardiac events and their potential drivers due to *Chkb* deficiency.** This study is the first to report that both heterozygous and homozygous *Chkb* (Choline kinase beta) deficiencies alter the cardiac lipid profile and membrane composition and are associated with cardiomyopathy. Chkb deficiency results in a significant decrease in the expression of *ANP*, its receptor *NPRA*, as well as ventricular conduction system markers (hyperpolarization-activated cyclic nucleotide-gated channel-4 [*HCN4*] and connexin 40 [*Cx40*]) in *Chkb*^+/−^ and *Chkb*^−/−^ mice. *ANP* expression has been shown to protect against the development of heart failure and is involved in the development of the embryonic ventricular conduction system. Defects in cardiac conduction system development in patients with congenital heart diseases can cause arrhythmias and may lead to sudden death. The decreased capacity of cardiac mitochondria from *Chkb*^−/−^ mice to utilize fatty acids for oxygen production results in accumulation of AcCa in cardiac muscle. Increased levels of long-chain AcCa have been associated with cardiovascular disease risk, heart failure, left ventricle remodeling and function proportional to disease stage and severity. Furthermore, the alterations in specific cardiac signaling pathways in *Chkb*-deficient hearts (decreased p-AKT, p-GSKβ, and p-AMPK) lead to defective response to extracellular stimuli and render the hearts more susceptible to cardiomyopathy.

This study is first to report that heterozygous and homozygous *Chkb* (choline kinase β) deficiencies are associated with cardiomyopathy, cardiac structural and functional defects, and an increased predisposition to arrythmia (Fig. 8). Although Chkb catalyzes the first step in the synthesis of the major phospholipid PC, the most notable change in lipid level observed in mice deficient in Chkb was an increase in the level of AcCa. Increased levels of long-chain AcCa have been associated with cardiovascular disease risk, heart failure, and left ventricle remodeling and function proportional to disease stage and severity (10, 11, 12, 13, 14, 15, 16, 17, 18, 19, 20, 21), phenotypes similar to those observed in the current study of *Chkb*-deficient mice. These clinical associations are supported by an extensive body of basic research from animal and cell models showing that long-chain AcCa exposure to cardiac muscle alters various cardiac excitation–contraction coupling processes and myocardial contractility. In fact, long-chain AcCa alters the activity of certain Na^+^, Ca^2+^, and K^+^-handling proteins to induce arrhythmias (39).

How the level of AcCa increases in the heart is not yet clear. We suspect that decreased capacity through the choline kinase step could decrease DG consumption *via* the downstream cholinephosphotransferase enzyme of the Kennedy pathway. This could back up that arm of the pathway such that fatty acids are shunted into AcCa for consumption (Fig. 4*A*); however, as we show that there is an inability to use AcCa as a substrate for mitochondrial β-oxidation this would result in AcCa accumulation. Testing this hypothesis will be an interesting avenue for future work.

*ANP* expression has been shown to protect against the development of heart failure (28, 29, 30), and we have previously shown that ANP signaling is involved in the development of the embryonic VCS (27). Defects in cardiac conduction system development in patients with congenital heart diseases can cause arrhythmias and may lead to sudden death (40). The decrease in ANP expression in *Chkb*-deficient heart that we report led to a decrease in expression of VCS markers. Decreased ANP and NPRA levels may alter the development of embryonic ventricular conduction system and render *Chkb*-deficient mice more susceptible to arrhythmia. The exact mechanism for reduced ANP and NPRA expression in *Chkb*-deficient mice is not clear. One possible mechanism is the alteration in membrane mechanosensory capacity of *Chkb*^−/−^ myocytes. Natriuretic peptides, including ANP, are secreted in response to the neurohumoral stimuli and/or stretching of atrial and ventricular walls and produce intense hypotensive effects *via* their diuretic, natriuretic, and vascular dilatory properties. Stimulation of secretion of ANP (and BNP) from the atria is mediated through mechanisms involving G proteins (G_q_ or G_o_ types) (41). Protein–lipid interactions are crucial for G protein–coupled receptor cellular localization, and consequently for signal transduction (42, 43, 44). Therefore, the alteration in lipid profile of *Chkb*-deficient cardiac myocytes might have disrupted the stretch-induced activation of the G protein–coupled receptors and downstream *ANP* expression. This will be interesting to further pursue.

Mitochondria occupy one-third of the cell volume in cardiac myocytes, and cardiac muscle has the highest oxygen consumption rate on a per unit weight basis (45). Mitochondrial fatty acid oxidation is the primary energy source utilized in the adult myocardium to produce energy and takes place *via* the electron transport chain located on the mitochondrial inner membrane (46). To use fatty acids as substrate for mitochondrial β-oxidation they must be first transported into mitochondria by a carnitine–acylcarnitine translocase through the inner mitochondrial membrane and transesterified back to acyl-CoA by carnitine palmitoyltransferase II. The released carnitine is shuttled back to the cytosol, and AcCa is transferred into the matrix. Here, we showed that cardiac mitochondria from *Chkb*^−/−^ mice have a decreased capacity to utilize fatty acids for oxygen consumption, consistent with the accumulation of AcCa we observed in cardiac muscle. The reduced mitochondrial oxygen consumption rate does not seem to be substrate dependent as we observed a reduction in OCR when we provided the mitochondria with succinate, which directly feeds into protein complex II and does not depend on the TCA cycle. Consistent with a generalized defect in electron transport chain function was our observation of an increase in deformed mitochondrial cristae (inner membrane) in Chkb-deficient mice.

Interesting, despite the fact that choline kinase catalyzes the first step in PC synthesis, a change in PC level does not seem to be the major metabolic driver behind the cardiomyopathy in *Chkb*-deficient mice. We determined that the level of the second choline kinase isoform present in mice, and humans, Chka, is maintained in cardiac muscle in *Chkb*-deficient mice. Previous studies determined that increased expression of *Chka* in *Chkb*-deficient mice can at least partially compensate for *Chkb* deficiency in muscle (47). In addition, PC can be replenished *via* exogenous PC supply as PC is imported into cells from serum *via* low-density lipoproteins. Indeed, enhanced expression of scavenger receptor-B1 and low-density lipoprotein receptor was previously observed in muscle of *Chkb*^−/−^ mice leading to enhanced PC uptake from plasma (48). Both of these processes may help normalize PC level in cardiac muscle of *Chkb*-deficient mice.

In summary, we found major similarities between the cardiac phenotypes observed in *CHKB* patients and *Chkb*-deficient mice. Using *Chkb*-deficient mice, our study added specificity and mechanism to help explain cardiac phenotypes observed in *CHKB* patients and introduce *Chkb*-deficient mice as a suitable model to further study the pathomechanism of cardiac defects in *CHKB* patients.

## Experimental procedures

### Mouse strains

All animal procedures were approved by the Dalhousie University’s Committee on laboratory animals in accordance with guidelines of the Canadian Council on Animal Care Guide to the Care and Use of Experimental Animals (CCAC, Ottawa, ON, Canada: vol. 1, second ed., 1993; vol. 2, 1984). Chkb mutant mice in C57BL/6J background were a kind gift from Professor Gregory A. Cox and were originally generated at the Jackson Laboratory (4). Male *Chkb*^*+/*−^ mice on the C57BL/6J background were crossed with female *Chkb*^*+/*−^ on the same background to generate *Chkb*^*+/+*^, *Chkb*^−*/*−^, and *Chkb*^*+/*−^ littermates. The mutation identified in *Chkb*^−*/*−^ mice is a 1.6-kb genomic deletion between exon 3 and intron 9 that results in expression of a truncated mRNA and the absence of Chkb protein expression (4).

### Mouse genotyping

The mutation identified in *Chkb*^−*/*−^ mice is a 1.6-kb genomic deletion between exon 3 and intron 9 (4). The AccuStart II Mouse Genotyping Kit was used to extract DNA from ear punches and to perform PCR analysis. A single genotyping program was used to amplify both the wildtype *Chkb* allele between exons 5 and 9 and the truncated *Chkb* allele between exons 2 and 10. The primers used for genotyping were purchased from Integrated DNA Technologies. The primer sequences to genotype wildtype are forward primer: 5′-GTG GGT GGC ACT GGC ATT TAT-3'; reverse primer: 5′-GTT TCT Accusant GTT CCT CTT CGG AGA-3' (amplicon size 753 bp). The primer sequences to genotype the mutants are forward primer: 5′-TAC CCA CGT ACC TCT GGC TTT T-3' reverse primer: 5′-GCT TTC CTG GAG GAC GTG AC-3′(amplicon size 486 bp). For each mouse, one PCR reaction was performed using both the primer sets. If two bands were observed, the mouse was characterized as heterozygous.

### Total RNA isolation, cDNA generation, and quantitative real-time RT–quantitative PCR

Isolated tissue samples were incubated overnight in prechilled RNAlater (Cat. no. R0901, Sigma-Aldrich) at 4 °C. Tissues were then homogenized in TRIzol reagent (Cat. no. 15596026, Invitrogen), and total RNA was isolated according to the manufacturer’s protocol. Nine hundred nanograms of total RNA was reverse transcribed using High-Capacity cDNA Reverse Transcription Kit (Cat. no. 4368814, Applied Biosystems). Quantitative real-time RT-PCR assays were performed on the LightCycler 96 (Roche Life Science) System using TaqMan Fast Advanced Master Mix (Cat. no. 4444557) and TaqManGene Expression Assays (Cat. no. 4331182, Thermo Fisher Scientific) for Cnsk2a2 (RRID: Mm01243455_m1) and Nd1 (Mm04225274_s1). Reactions were run in triplicate. Quantitative real-time RT-PCR for ANP, NPRA, Gapdh, Cx40, and HCN4 was performed according to our previous publications (27, 49).

### Immunofluorescent microscopy

Cryosections (10 μm) from adult hearts were fixed with 4% w/v paraformaldehyde (pH 7.4) for 5 min at room temperature and were then permeabilized in 0.1% v/v Triton X-100 (Sigma) for 4 min. Following this, sections were covered in blocking buffer solution (10% v/v goat serum [Gibo] and 1% w/v bovine serum albumin [BSA; Thermo Fisher Scientific] in PBS) for 1 h at room temperature. After 1 h, blocking buffer solution was removed and replaced with blocking buffer containing primary antibodies for Connexin40 (Cx40) (1:50, Alpha Diagnostics, Cat# IGG1 CX40A), Sarcomeric myosin (MF20) (1:50, Developmental Studies Hybridoma Bank, Cat# MF-20). Slides were then washed with PBS three times for 3 min each and were then incubated with secondary goat anti-mouse antibody conjugated to Alexa Fluor 488 (1:200, Invitrogen) and goat anti-rabbit antibody conjugated to Alexa Fluor 555 (1:200, Invitrogen) in blocking buffer for 1 hour. Nuclei were counterstained by immersion of a solution containing 1 μg/ml Hoechst 33258 (Sigma) in PBS. Slides were mounted with 0.1% propyl gallate (Sigma) solution (0.1% w/v propyl gallate and 50% v/v glycerol [Thermo Fisher Scientific] in PBS) and were observed under a laser scanning confocal microscope (Zeiss LSM 710).

### Transmission electron microscopy

For transmission electron microscopy (TEM) analysis, ∼5 × 5-mm cubes of cardiac muscles were fixed with 2.5% glutaraldehyde diluted with 0.1 M sodium cacodylate buffer and postfixed with 1% osmium tetroxide in Millonig’s buffer solution for 2 h, dehydrated, and embedded in epon araldite resin. Ultrathin sections were stained with 2% uranyl acetate for 30 min and lead citrate for 4 min and viewed with a JEOL JEM 1230 Transmission Electron Microscope at 80 kV. Images were captured using a Hamamatsu ORCA-HR digital camera. Three mice per genotype for each timepoint were evaluated. The mitochondrial content was determined from the images at 100,00× magnification using Image J software and calculated as mitochondria count/field by blinded investigators. Point counting was used to estimate mitochondrial volume density based on standard stereological methods (50). Grid sizes of 165 nm × 165 nm were used to estimate mitochondria volume density. Mitochondria volume density was calculated by dividing the points assigned to mitochondria by the total number of points counted inside the muscle.

### Western blot analysis and quantification

The cardiac muscle tissue (∼100 mg) was homogenized with a steel bead in 1 ml of cold RIPA buffer containing 1× Proteinase Inhibitor Mix (complete Protease Inhibitor Cocktail, Roche, Cat. no.11 697 498 001), 1× PhosStop (Roche, Cat. no.04 906 845 001) using a TissueLyser II instrument (Qiagen) set at 30 strokes/s for 2 to 4 min. Based on BCA protein quantification results, all samples were adjusted to the final concentration of 2 μg/μl and heat denatured for 5 min at 99 °C in 2× Laemmli buffer. Proteins were separated by SDS-PAGE and transferred to nitrocellulose membranes. The membranes were incubated in Odyssey blocking solution for 1 h. Total proteins were detected by probing the membranes with appropriate primary antibodies overnight at 4 °C. The following antibodies were used: Chkα (1:1000, Abcam Cat#ab88053), Chkβ (1:250, Santa Cruz, Cat#398957), Gapdh (1:1000, Cell Signaling, Cat#398957), AKT (1:1000, Cell Signaling, Cat#4691), Phospho-AKT(Ser473) (Cell Signaling, Cat#:4060), AMPKα (1:1000, Cell Signaling, Cat#2532), Phospho-AMPKα (Thr172) (1:1000, Cell Signaling, Cat#2535), Phospho-GSK-3β (Ser9) (1:1000, Cell Signaling, Cat#9336), GSK-3β (1:1000, Cell Signaling, Cat#9315), α-actinin (1:1000, Santa Cruz, Cat#sc17829), Talin (1:1000, Cell Signaling, Cat#4777), Vinculin (1:1000, Abcam, Cat#ab18058), Lamin A/C (1:1000, Cell Signaling, Cat#2032). Proteins were visualized with goat anti-rabbit IRDye-800- or -680-secondary antibodies (LI-COR Biosciences) using an Odyssey imaging system, and band density was evaluated using FIJI (NIH).

### Isolation of mitochondria from mouse hearts

The mitochondrial isolation protocol was standardized in our laboratory and developed from previously published protocols (51, 52). The heart was extracted, washed in PBS, and minced in 2 ml of MSHE + BSA (70 mM sucrose, 210 mM mannitol, 5 mM Hepes, 1 mM EGTA, and 0.5% (w/v) fatty acid–free BSA, pH 7.2) at 4 °C. All subsequent steps were performed on ice. The tissue was homogenized using a glass Teflon Dounce homogenizer for 6 to 7 strokes. The homogenate was centrifuged at 800*g* for 10 min at 4 °C, and the supernatant was filtered by a prewet 40-μm mesh filter into the 50-ml conical centrifuge tube on ice. This step was repeated by a 10-μm mesh filter (pluriStrainer, Leipzig, Germany). The filtrate was then centrifuged at 8000*g* for 10 min at 4 °C. After removal of the supernatant, the final pellet was resuspended in 120 μl of MSHE + BSA. A portion of this suspension was further centrifuged at 10,000*g* for 10 min and the pellet was suspended in water for protein quantification by BCA.

### Seahorse analysis of mitochondrial function

Oxygen consumption rate (OCR) was measured as described (53) using a Seahorse XF24 extracellular flux analyzer (Seahorse Biosciences). Sensor calibration was performed according to the manufacturer’s instructions. Isolated mitochondria were diluted to the desired concentration required for plating (3.5–4 μg) and spun at 2000*g* for 20 min at 4 °C. After centrifugation, 155 μl of prewarmed mitochondrial assay solution (MAS) (70 mM sucrose, 220 mM mannitol, 10 mM KH_2_PO_4_, 5 mM MgCl_2_, 2 mM Hepes, 1 mM EGTA, and 0.2% (w/v) fatty acid–free BSA, pH 7.2 at 37 °C) + succinate (10 mM) + rotenone (4 μM) or MAS+ palmitoyl carnitine (80 μM)/malate (0.5 mM) was added to each well. ADP, 4 mM, was also added to the wells prior to the assay. For all assays, mitochondrial function was probed by the sequential addition of oligomycin (4 μM), FCCP (carbonyl cyanide 4-(trifluoromethoxy) phenylhydrazone; 6 μM), rotenone (2 μM), and antimycin A (12.5 μM), all final concentrations. Three measurements were performed for each condition. All experiments were normalized to total protein as determined by a BCA protein quantitation assay.

### Electrocardiography

Animals were anesthetized using 1% to 1.5% isoflurane, and the ECG signals were obtained using a bipolar three-electrode three-lead system and analyzed *via* Lab Chart 7 v.7.3.7 software (AD Instruments Inc). The positive and negative leads were placed under the skin of the left and right pectoral muscle of mice, and the ground lead was placed under the skin of the left hind limb. During ECG analysis, mice body temperatures were maintained at 37 °C using a small animal heating plate. For each animal, the ECG signal was recorded for approximately 15 min and at least 10 beats were averaged to determine the heart rate, PR, RR, QRS, QT, and P duration at baseline. After a stable baseline was obtained, isoproterenol (Isuprel, 1.5 mg/kg) was injected i.p. and the ECG recordings were continued for 15 min. Traces were then examined for the presence of arrhythmic beats, premature ventricular contractions, and ventricular tachycardia were quantified as number of beats/recordings.

### Echocardiography

Mice were anesthetized with inhaled isoflurane and placed on a heated imaging platform. The temperature and heart rate of the mouse were continuously monitored to minimize physiological variation. Transthoracic echocardiography was performed using a high-resolution transducer and a Visual Sonics Vevo 2100 Ultra High-Frequency Imaging Platform (FUJIFILM). Cardiac function and structure were assessed by measuring two-dimensional M-Mode images from the parasternal short and long axis at the level of the midpapillary muscle and the parasternal long axis. Recordings were then analyzed using the Vevo 2100 Software as per the company’s direction provided in their training manual. From these measurements the following calculations were completed: cardiac output, ejection fraction, fractional shortening, stroke volume, IVSd, LVIDd LVPWd, IVSs, LVIDs, LVPWs, LA, Ao Sinus, E/A ratio, A’/E′ velocities ratio, E’/A′ velocities ratio, and MV E/E′ ratio. Animals were allotted time to recover prior to being returned to their colony.

### Quantification and statistical analysis

All experiments were repeated three or more times. Data are presented as mean ± SEM or mean ± SD, as appropriate. For comparison of two groups the two-tailed Student’s *t* test was used unless otherwise specified. Comparison of more than two groups was done by one-way ANOVA followed by the Tukey’s multiple comparison test. *p* values <0.05 were considered significant.

### Lipid extraction

We performed lipid extractions using the modified Bligh and Dyer extraction for LC-MS analysis of lipids protocol 40. All reagents were of LC-MS grade. Briefly, the cardiac muscle tissue (∼10 mg) was homogenized with a steel bead in 1 ml of cold 0.1 M HCl:methanol (1:1, v/v) using a TissueLyser II instrument (Qiagen) set at 30 strokes/s for 2 to 4 min. Based on BCA protein quantification results, all samples were adjusted to the final concentration of 700 μg/ml and spiked with 10 μl of internal standard (Avanti Polar Lipids Inc; Catalog Number-330707). Chloroform, 500 μl, was added to each sample, vortexed for 30 min, and centrifuged to separate phases (5 min at 6000 rpm). The bottom organic phase was transferred into a new Eppendorf tube and dried under a nitrogen stream. Samples were stored at −80 °C until ready for analysis.

### Ultra-HPLC method for lipid analysis

The Accucore C30 column (250.2.1 mm I.D., particle size: 2.8 μm) was obtained from Thermo Fisher Scientific. The mobile phase system consisted of solvent A (acetonitrile:water 60:40 v/v) and solvent B (isopropanol:acetonitrile:water 90:10:1 v/v), both containing 10 mM ammonium formate and 0.1% formic acid. C30-RPLC separation was carried out at 30 °C (column oven temperature) with a flow rate of 0.2 ml/min, and 10 μl of the lipid extraction suspended in the mobile phase solvents mixtures (A:B, 70:30) was injected onto the column. The following system gradient was used for separating the lipid classes and molecular species: 30% solvent B for 3 min; then solvent B increased to 50% over 6 min, then to 70% B in 6 min, then kept at 99% B for 20 min, and finally the column was re-equilibrated to starting conditions (30% solvent A) for 5 min prior to each new injection.

### High-resolution tandem mass spectrometry and lipidomics

Lipid analyses were carried out using a Q-Exactive Orbitrap mass spectrometer controlled by X-Calibur software 4.0 (Thermo Scientific) with an acquisition HPLC system. The following parameters were used for the Q-Exactive mass spectrometer: sheath gas, 40; auxiliary gas, 5; ion spray voltage, 3.5 kV; capillary temperature, 250 °C; mass range, 200 to 2000 *m/z*; full scan mode at a resolution of 70,000 *m/z*; top-1 *m/z* and collision energy of 35 (arbitrary unit); isolation window, 1 *m/z*; automatic gain control target, 1e5. The instrument was externally calibrated to 1 ppm using ESI negative and positive calibration solutions (Thermo Scientific). Tune parameters were optimized using a mixture of lipid standards (Avanti Polar Lipids) in both negative and positive ion modes. Thermo Scientific LipidSearch software version 4.2 was used for lipid identification and quantitation. First, the individual data files were searched for product ion tandem mass spectrometry spectra of lipid precursor ions. Tandem mass spectrometry fragment ions were predicted for all precursor adduct ions measured within ±5 ppm. The product ions that matched the predicted fragment ions within a ±5 ppm mass tolerance was used to calculate a match score, and those candidates providing the highest quality match were determined. Next, the search results from the individual positive or negative ion files from each sample group were aligned within a retention time window (±0.2 min) and the data were merged for each annotated lipid.

### Data cleanup and statistical analysis of lipids

Lipid concentrations extracted from the LipidSearch software were further analyzed with an in-house script using the R programming language (available upon request). The data were filtered to exclude any peak concentration estimates with a signal to noise ratio (SNR parameter) of less than 2.0 or a peak quality score (PQ parameter) of less than 0.8. If this exclusion resulted in the removal of two observations within a biological triplicate, the remaining observation was also excluded. The individual concentrations were then gathered together by lipid identity (summing together the concentration of multiple mass spectrometry adducts where these adducts originated from the same molecular source and averaging together biological replicates) and grouped within the broader categories. The result was nine groups containing multiple lipid concentrations corresponding to specific lipid identities, which were then compared between wildtype and KO samples using a (paired, nonparametric) Wilcoxon signed-rank test at an overall significance level of 5% (using the Bonferroni correction to account for the large number of tests performed). As the Bonferroni correction is fairly conservative, significant differences are reported at both precorrection (∗) and postcorrection (∗∗∗) significance levels.

## Data availability

The authors confirm that the data supporting the findings of this study are available within the article. Primary data are available from the corresponding author upon reasonable request.

## Conflict of interest

The authors declare that they have no conflicts of interest with the contents of this article.

## References

[1] Molnar P., Hickman J.J. (2014). Modeling of action potential generation in NG108-15 cells. Methods Mol. Biol..

[2] Whited A.M., Johs A. (2015). The interactions of peripheral membrane proteins with biological membranes. Chem. Phys. Lipids.

[3] Vance J.E. (2015). Phospholipid synthesis and transport in mammalian cells. Traffic.

[4] Sher R.B., Aoyama C., Huebsch K.A., Ji S., Kerner J., Yang Y., Frankel W.N., Hoppel C.L., Wood P.A., Vance D.E., Cox G.A. (2006). A rostrocaudal muscular dystrophy caused by a defect in choline kinase beta, the first enzyme in phosphatidylcholine biosynthesis. J. Biol. Chem..

[5] Mitsuhashi S., Ohkuma A., Talim B., Karahashi M., Koumura T., Aoyama C., Kurihara M., Quinlivan R., Sewry C., Mitsuhashi H., Goto K., Koksal B., Kale G., Ikeda K., Taguchi R. (2011). A congenital muscular dystrophy with mitochondrial structural abnormalities caused by defective de novo phosphatidylcholine biosynthesis. Am. J. Hum. Genet..

[6] Chan S.H., Ho R.S., Khong P.L., Chung B.H., Tsang M.H., Yu M.H., Yeung M.C., Chan A.O., Fung C.W. (2020). Megaconial congenital muscular dystrophy: Same novel homozygous mutation in CHKB gene in two unrelated Chinese patients. Neuromuscul. Disord..

[7] Haliloglu G., Talim B., Sel C.G., Topaloglu H. (2015). Clinical characteristics of megaconial congenital muscular dystrophy due to choline kinase beta gene defects in a series of 15 patients. J. Inherit. Metab. Dis..

[8] Quinlivan R., Mitsuahashi S., Sewry C., Cirak S., Aoyama C., Mooore D., Abbs S., Robb S., Newton T., Moss C., Birchall D., Sugimoto H., Bushby K., Guglieri M., Muntoni F. (2013). Muscular dystrophy with large mitochondria associated with mutations in the CHKB gene in three British patients: Extending the clinical and pathological phenotype. Neuromuscul. Disord..

[9] Vanlander A.V., Muino Mosquera L., Panzer J., Deconinck T., Smet J., Seneca S., Van Dorpe J., Ferdinande L., Ceuterick-de Groote C., De Jonghe P., Van Coster R., Baets J. (2016). Megaconial muscular dystrophy caused by mitochondrial membrane homeostasis defect, new insights from skeletal and heart muscle analyses. Mitochondrion.

[10] Kalim S., Clish C.B., Wenger J., Elmariah S., Yeh R.W., Deferio J.J., Pierce K., Deik A., Gerszten R.E., Thadhani R., Rhee E.P. (2013). A plasma long-chain acylcarnitine predicts cardiovascular mortality in incident dialysis patients. J. Am. Heart Assoc..

[11] Cheng M.L., Wang C.H., Shiao M.S., Liu M.H., Huang Y.Y., Huang C.Y., Mao C.T., Lin J.F., Ho H.Y., Yang N.I. (2015). Metabolic disturbances identified in plasma are associated with outcomes in patients with heart failure: Diagnostic and prognostic value of metabolomics. J. Am. Coll. Cardiol..

[12] Milani-Nejad N., Janssen P.M. (2014). Small and large animal models in cardiac contraction research: Advantages and disadvantages. Pharmacol. Ther..

[13] Lai L., Leone T.C., Keller M.P., Martin O.J., Broman A.T., Nigro J., Kapoor K., Koves T.R., Stevens R., Ilkayeva O.R., Vega R.B., Attie A.D., Muoio D.M., Kelly D.P. (2014). Energy metabolic reprogramming in the hypertrophied and early stage failing heart: A multisystems approach. Circ. Heart Fail..

[14] Rizza S., Copetti M., Rossi C., Cianfarani M.A., Zucchelli M., Luzi A., Pecchioli C., Porzio O., Di Cola G., Urbani A., Pellegrini F., Federici M. (2014). Metabolomics signature improves the prediction of cardiovascular events in elderly subjects. Atherosclerosis.

[15] Zordoky B.N., Sung M.M., Ezekowitz J., Mandal R., Han B., Bjorndahl T.C., Bouatra S., Anderson T., Oudit G.Y., Wishart D.S., Dyck J.R., Alberta H. (2015). Metabolomic fingerprint of heart failure with preserved ejection fraction. PLoS One.

[16] Ahmad T., Kelly J.P., McGarrah R.W., Hellkamp A.S., Fiuzat M., Testani J.M., Wang T.S., Verma A., Samsky M.D., Donahue M.P., Ilkayeva O.R., Bowles D.E., Patel C.B., Milano C.A., Rogers J.G. (2016). Prognostic implications of long-chain acylcarnitines in heart failure and reversibility with mechanical circulatory support. J. Am. Coll. Cardiol..

[17] Hunter W.G., Kelly J.P., McGarrah R.W., Khouri M.G., Craig D., Haynes C., Ilkayeva O., Stevens R.D., Bain J.R., Muehlbauer M.J., Newgard C.B., Felker G.M., Hernandez A.F., Velazquez E.J., Kraus W.E. (2016). Metabolomic profiling identifies novel circulating biomarkers of mitochondrial dysfunction differentially elevated in heart failure with preserved *versus* reduced ejection fraction: Evidence for shared metabolic impairments in clinical heart failure. J. Am. Heart Assoc..

[18] Lanfear D.E., Gibbs J.J., Li J., She R., Petucci C., Culver J.A., Tang W.H.W., Pinto Y.M., Williams L.K., Sabbah H.N., Gardell S.J. (2017). Targeted metabolomic profiling of plasma and survival in heart failure patients. JACC Heart Fail..

[19] Ruiz M., Labarthe F., Fortier A., Bouchard B., Thompson Legault J., Bolduc V., Rigal O., Chen J., Ducharme A., Crawford P.A., Tardif J.C., Des Rosiers C. (2017). Circulating acylcarnitine profile in human heart failure: A surrogate of fatty acid metabolic dysregulation in mitochondria and beyond. Am. J. Physiol. Heart Circ. Physiol..

[20] Elmariah S., Farrell L.A., Furman D., Lindman B.R., Shi X., Morningstar J.E., Rhee E.P., Gerszten R.E. (2018). Association of acylcarnitines with left ventricular remodeling in patients with severe aortic stenosis undergoing transcatheter aortic valve replacement. JAMA Cardiol..

[21] Verdonschot J.A.J., Wang P., Van Bilsen M., Hazebroek M.R., Merken J.J., Vanhoutte E.K., Henkens M., Van Den Wijngaard A., Glatz J.F.C., Krapels I.P.C., Brunner H.G., Heymans S.R.B., Bierau J. (2020). Metabolic profiling associates with disease severity in nonischemic dilated cardiomyopathy. J. Card. Fail..

[22] Fillmore N., Mori J., Lopaschuk G.D. (2014). Mitochondrial fatty acid oxidation alterations in heart failure, ischaemic heart disease and diabetic cardiomyopathy. Br. J. Pharmacol..

[23] Daum G. (1985). Lipids of mitochondria. Biochim. Biophys. Acta.

[24] Weibel E.R., Gehr P., Cruz-Orive L.M., Muller A.E., Mwangi D.K., Haussener V. (1981). Design of the mammalian respiratory system. IV Morphometric estimation of pulmonary diffusing capacity; critical evaluation of new sampling method. Respir. Physiol..

[25] Mahmaljy H., Yelamanchili V.S., Singhal M. (2021).

[26] Sergeeva I.A., Christoffels V.M. (2013). Regulation of expression of atrial and brain natriuretic peptide, biomarkers for heart development and disease. Biochim. Biophys. Acta.

[27] Govindapillai A., Hotchkiss A., Baguma-Nibasheka M., Rose R.A., Miquerol L., Smithies O., Maeda N., Pasumarthi K.B.S. (2018). Characterizing the role of atrial natriuretic peptide signaling in the development of embryonic ventricular conduction system. Sci. Rep..

[28] Stevens T.L., Burnett J.C., Kinoshita M., Matsuda Y., Redfield M.M. (1995). A functional role for endogenous atrial natriuretic peptide in a canine model of early left ventricular dysfunction. J. Clin. Invest..

[29] Wang D., Gladysheva I.P., Fan T.H., Sullivan R., Houng A.K., Reed G.L. (2014). Atrial natriuretic peptide affects cardiac remodeling, function, heart failure, and survival in a mouse model of dilated cardiomyopathy. Hypertension.

[30] Yasuno S., Usami S., Kuwahara K., Nakanishi M., Arai Y., Kinoshita H., Nakagawa Y., Fujiwara M., Murakami M., Ueshima K., Harada M., Nakao K. (2009). Endogenous cardiac natriuretic peptides protect the heart in a mouse model of dilated cardiomyopathy and sudden death. Am. J. Physiol. Heart Circ. Physiol..

[31] Miquerol L., Moreno-Rascon N., Beyer S., Dupays L., Meilhac S.M., Buckingham M.E., Franco D., Kelly R.G. (2010). Biphasic development of the mammalian ventricular conduction system. Circ. Res..

[32] Muallem S., Chung W.Y., Jha A., Ahuja M. (2017). Lipids at membrane contact sites: Cell signaling and ion transport. EMBO Rep..

[33] Sunshine H., Iruela-Arispe M.L. (2017). Membrane lipids and cell signaling. Curr. Opin. Lipidol..

[34] Fujio Y., Nguyen T., Wencker D., Kitsis R.N., Walsh K. (2000). Akt promotes survival of cardiomyocytes *in vitro* and protects against ischemia-reperfusion injury in mouse heart. Circulation.

[35] Taniyama Y., Walsh K. (2002). Elevated myocardial Akt signaling ameliorates doxorubicin-induced congestive heart failure and promotes heart growth. J. Mol. Cell. Cardiol..

[36] Walsh K. (2006). Akt signaling and growth of the heart. Circulation.

[37] Dyck J.R., Lopaschuk G.D. (2006). AMPK alterations in cardiac physiology and pathology: Enemy or ally?. J. Physiol..

[38] Gallo S., Vitacolonna A., Bonzano A., Comoglio P., Crepaldi T. (2019). ERK: A key player in the pathophysiology of cardiac hypertrophy. Int. J. Mol. Sci..

[39] Aitken-Buck H.M., Krause J., Zeller T., Jones P.P., Lamberts R.R. (2020). Long-chain acylcarnitines and cardiac excitation-contraction coupling: Links to arrhythmias. Front. Physiol..

[40] Park D.S., Fishman G.I. (2011). The cardiac conduction system. Circulation.

[41] de Bold A.J. (2011). Thirty years of research on atrial natriuretic factor: Historical background and emerging concepts. Can. J. Physiol. Pharmacol..

[42] Escriba P.V., Ozaita A., Ribas C., Miralles A., Fodor E., Farkas T., Garcia-Sevilla J.A. (1997). Role of lipid polymorphism in G protein-membrane interactions: Nonlamellar-prone phospholipids and peripheral protein binding to membranes. Proc. Natl. Acad. Sci. U. S. A..

[43] Desai A.J., Miller L.J. (2018). Changes in the plasma membrane in metabolic disease: Impact of the membrane environment on G protein-coupled receptor structure and function. Br. J. Pharmacol..

[44] Torres M., Rossello C.A., Fernandez-Garcia P., Llado V., Kakhlon O., Escriba P.V. (2020). The implications for cells of the lipid switches driven by protein-membrane interactions and the development of membrane lipid therapy. Int. J. Mol. Sci..

[45] Schaper J., Meiser E., Stammler G. (1985). Ultrastructural morphometric analysis of myocardium from dogs, rats, hamsters, mice, and from human hearts. Circ. Res..

[46] Kolwicz S.C., Purohit S., Tian R. (2013). Cardiac metabolism and its interactions with contraction, growth, and survival of cardiomyocytes. Circ. Res..

[47] Sayed-Zahid A.A., Sher R.B., Sukoff Rizzo S.J., Anderson L.C., Patenaude K.E., Cox G.A. (2019). Functional rescue in a mouse model of congenital muscular dystrophy with megaconial myopathy. Hum. Mol. Genet..

[48] Wu G., Sher R.B., Cox G.A., Vance D.E. (2009). Understanding the muscular dystrophy caused by deletion of choline kinase beta in mice. Biochim. Biophys. Acta.

[49] Hotchkiss A., Feridooni T., Baguma-Nibasheka M., McNeil K., Chinni S., Pasumarthi K.B. (2015). Atrial natriuretic peptide inhibits cell cycle activity of embryonic cardiac progenitor cells *via* its NPRA receptor signaling axis. Am. J. Physiol. Cell Physiol..

[50] Weibel E.R. (1981). Stereological methods in cell biology: Where are we--where are we going?. J. Histochem. Cytochem..

[51] Schnaitman C., Greenawalt J.W. (1968). Enzymatic properties of the inner and outer membranes of rat liver mitochondria. J. Cell Biol..

[52] Preble J.M., Pacak C.A., Kondo H., MacKay A.A., Cowan D.B., McCully J.D. (2014). Rapid isolation and purification of mitochondria for transplantation by tissue dissociation and differential filtration. J. Vis. Exp..

[53] Boutagy N.E., Rogers G.W., Pyne E.S., Ali M.M., Hulver M.W., Frisard M.I. (2015). Using isolated mitochondria from minimal quantities of mouse skeletal muscle for high throughput microplate respiratory measurements. J. Vis. Exp..

